# Thermal Ablative Therapies and Immune Checkpoint Modulation: Can Locoregional Approaches Effect a Systemic Response?

**DOI:** 10.1155/2016/9251375

**Published:** 2016-03-08

**Authors:** Amol Mehta, Rahmi Oklu, Rahul A. Sheth

**Affiliations:** ^1^University of Pittsburgh, Pittsburgh, PA 15213, USA; ^2^Mayo Clinic, Scottsdale, AZ 85259, USA; ^3^MD Anderson Cancer Center, Houston, TX 77030, USA

## Abstract

Percutaneous image-guided ablation is an increasingly common treatment for a multitude of solid organ malignancies. While historically these techniques have been restricted to the management of small, unresectable tumors, there is an expanding appreciation for the systemic effects these locoregional interventions can cause. In this review, we summarize the mechanisms of action for the most common thermal ablation modalities and highlight the key advances in knowledge regarding the interactions between thermal ablation and the immune system.

## 1. Introduction

Percutaneous image-guided ablation procedures are the cornerstones in the management of numerous solid organ malignancies [1]. While ablation has historically been considered analogous to surgical resection insofar as it results in focal eradication of tumor, there are several important differences. The most substantial distinction is that ablation causes cell death* in situ*; this exposes previously shielded tumor antigens to the immune system and can incite an inflammatory response that may lead to either distant tumor growth suppression [2] or acceleration [3]. Both positive (remote tumor regression) and negative (remote tumor growth) outcomes have been observed in preclinical and clinical cases following ablation. In this review, we summarize the current data regarding systemic responses to ablative therapies, emphasizing the key cellular mechanisms and role of the immune system. We additionally highlight examples of synergy between immune checkpoint modulation and thermal ablation reported in preclinical studies to motivate further investigations in this potentially transformative approach to cancer therapy.

## 2. Mechanisms of Action for Clinical Ablative Therapies

Multiple minimally invasive thermal ablation technologies are used in clinical practice today, including radiofrequency ablation (RFA), microwave ablation (MWA), laser interstitial thermal therapy (LITT), and high intensity focused ultrasound (HIFU). While each modality accomplishes the local deposition of energy via vastly different approaches, several generalities regarding these heat-based technologies can be made [2]. Of note, cryoablation, another commonly used ablation technique, effects cell death via mechanisms disparate from these heat-based methods and is discussed separately. The extent of cellular damage caused by heat-based ablative therapies depends on three factors: the amount of energy applied, the rate of energy delivery, and the target tissue's intrinsic thermal sensitivity [4]. Importantly, tumor tissue is believed to be more thermosensitive than normal tissue; this may be due to relatively increased cellular density, fewer interstitial vascular and lymphatic channels to dissipate heat, and the hypoxic/acidic tumor microenvironment [5–8].

The degree of hyperthermia during an ablation procedure varies in both space and time. With all heat-based ablation modalities, the targeted tissue can be divided into two zones. The central zone refers to the area of the tumor into which energy is directly deposited by the ablation device. In this zone, lethal hyperthermia is typically consistently achieved, and cell death via coagulative necrosis occurs. The necrotic debris that accumulates in this zone serves as a vital reservoir for tumor antigens that gradually drain to regional lymph nodes [9, 10].

The central zone is surrounded by a peripheral zone, composed of tissue into which heat is transferred via conduction from the central zone. In this zone, lethal hyperthermia may not be consistently reached, and cell death may occur via apoptosis due to heat-mediated lysosomal activation or mitochondrial damage rather than necrosis [11]; alternatively, cell recovery may occur. Indeed, apoptosis likely plays a key role in cell death in the peripheral heating zone [11]. Cell death by apoptosis is widely considered to be immunosuppressive and therefore opposes the development of abscopal effects [12]. As such, the balance of tumor necrosis to apoptosis is a critical yet poorly characterized factor in the immune response following ablative therapies.

A limitation that is shared by all heat-based modalities that primarily affects treatment efficacy within the peripheral zone is the heat sink effect [13]. This effect refers to the dissipation of thermal energy from the ablation zone by blood flowing within an adjacent blood vessel; tumoral tissue in continuity with blood vessels thus may not reach cytotoxic temperatures. As the relative proportion of central to peripheral zone within the overall ablation zone varies from one modality to another, the significance of the heat sink effect is modality dependent. This effect has been most extensively evaluated with RFA. Preclinical studies have shown that the heat sink effect can be consistently demonstrated with blood vessels greater than 3 mm in size. This effect has also been observed in the clinical setting, with higher rates of recurrence in tumors adjacent to large blood vessels [14].

A commonly used threshold for lethal hyperthermia is 50°C; that is, for temperatures above 50°C, coagulative necrosis due to protein denaturation occurs essentially immediately, while for temperatures just below 50°C, sustained exposure is required for cell death [4, 5, 15]. At lethal hyperthermic temperatures, cell death also occurs through mechanisms involving mitochondrial dysfunction [4] and suppression of DNA replication [16]. If immediate cell death is not achieved, a delayed response due to heat-induced cellular damage can be observed after the return to normothermic conditions. In this setting, cell death can occur through apoptosis, possibly driven by vascular thrombosis resulting in tissue ischemia or by reperfusion injury [4, 17].

### 2.1. Radiofrequency Ablation

Radiofrequency ablation requires the insertion of one or more RF applicators within a target lesion under imaging guidance, typically ultrasound or computed tomography (CT). One or more grounding pads is also placed on the patient at a site remote to the applicator, such as the patient's back or thigh. An electrical circuit is thus established within the patient, and when a high power, alternating current is generated by a power source between the applicators and the grounding pads, energy is deposited via frictional heating of ions that oscillate due to the alternating current. Radiofrequency ablation achieves temperatures of 60–100°C within the central zone. At temperatures of above 100°C, tissue charring occurs, at which point impedance increases by orders of magnitude decreasing electrical conduction and rendering the technique less effective. The electrical conductivity of the tissue being ablated, therefore, plays an important role in the degree of heat generation. As Goldberg et al. estimate to a first-order approximation, RF-induced heat transfer can be simplified as follows: “coagulation necrosis = energy deposited × local tissue interactions − heat lost” [18].

Of all the heat-based ablation technologies, RFA is in principle the most susceptible to the heat sink effect [13]. However, in a clinical trial comparing the efficacy of RFA to MWA for hepatocellular carcinoma, no significant difference was seen for complete response, local tumor recurrence, and overall survival rates [19].

### 2.2. Microwave Ablation

Microwave ablation requires the direct insertion of an antenna or antennae into a target lesion using imaging guidance and thus from a procedural standpoint is very similar to RFA. However, unlike RFA, MWA does not require the establishment of an electrical circuit. Instead, microwaves are emitted from the antenna into the surrounding tissue at the resonance frequency of water molecules, resulting in oscillation of these molecules and subsequent release of this kinetic energy as heat [20]. A newer technology than RFA, MWA, offers several technical advantages. Since MWA does not rely on electrical currents or conduction through tissue, temperatures > 100°C can be and often are achieved in the central zone. This allows for larger zones of ablation and shorter treatment times, as the central zone is larger relative to RFA, and conduction of heat from the central zone results in a larger peripheral zone and an overall larger volume of lethal hyperthermia. The larger central zone also lessens (but does not eradicate) the heat sink effect. The size of the ablation zone with MWA can be harder to predict than RFA and can lead to overtreatment and damage to adjacent off-target structures.

### 2.3. Cryoablation

In contradistinction to the above modalities, cryoablation results in local cell death via the removal of heat. As with heat-based modalities, cryoablation requires the image-guided insertion of a specialized cryoprobe into the target lesion. A room temperature gas, typically argon, is pumped into the cryoprobe. When this gas reaches the tip of the cryoprobe, it is forced through a narrow throttle and then allowed to rapidly expand. Through a mechanism known as the Joule-Thompson effect, this rapid expansion results in a decrease in temperature around the cryoprobe [21]. Heat is transferred via conduction and convection out of the surrounding tissues, and thus a zone of lethal hypothermia is created. During a cryoablation procedure, several cycles of freezing followed by thawing are performed. While cryoablation can lower interstitial temperatures to −160°C, temperatures required for cell typically range from −20 to −40°C.

Cell death in cryoablation is dependent on four variables: the rate of cooling, the minimum temperature achieved, the duration of time at the minimum temperature, and the rate of thawing [21, 22]. Cryoablation causes both direct and indirect cell death. In the former circumstance, cell injury occurs from dehydration as a result of freezing. Due to high intracellular osmolality, water freezes in the extracellular compartment before the intracellular compartment; the resultant osmotic gradient drives fluid out of the cells. During the thaw phase of a cryoablation cycle, the reversal of the osmotic gradient causes rapid influx of water, leading to swelling and cell rupture. Moreover, if cooling occurs rapidly, intracellular ice crystals can form, which physically damage organelles and the plasma membrane; during the thaw phase, intracellular ice may actually grow, as the influx of extracellular fluid lowers intracellular osmolality [21, 23]. Cell death via direct cooling injury results in coagulative necrosis. Alternatively, for cells that are not exposed to lethal levels of hypothermia, cold-induced injury to mitochondria can result in delayed apoptosis-mediated cell death; this typically occurs at the periphery of the cryoablation zone. The balance between coagulative necrosis and apoptosis can have important implications with regard to immunomodulation, as described below. Indirect methods of cell death are mediated by injury to blood vessels due to cold-induced endothelial cell dysfunction, initiating a cascade of platelet aggregation, microthrombus formation, and ischemia.

## 3. Requirements for Acquired Immune System Activation

When cell death occurs, the “first responders” are typically representatives of the innate immune system, including neutrophils, macrophages, and natural killer (NK) cells. This is followed by the more robust and sustained acquired immune response. However, there are four requirements for activating the acquired immune system: antigen presentation, antigen recognition by T-cells, interaction of costimulatory molecules, and the presence of danger signals [24]. Cell necrosis results in the spillage of intracellular antigens that were previously invisible from the immune system. These antigens are acquired by antigen presenting cells, of which dendritic cells (DCs) are the most important. Dendritic cells then localize to regional lymph nodes, where they present antigens to T-cells through major histocompatibility complex (MHC) molecules. Recognition of the antigen by the T-cell is necessary but not sufficient for T-cell proliferation and survival. Without concomitant costimulation, T-cells may undergo anergy and cell death. Costimulation refers to interactions between non-antigen-specific markers on the DC and T-cell, specifically CD28 on T-cells and the B7 molecules (also known as CD80 and CD86) on DCs. Alternatively, binding of the inhibitory signaling molecule CTLA-4 on the T-cell's surface with CD80 and CD86 functions as an “off” switch, inactivating the T-cell [25]. Finally, for DCs to activate T-cells, they themselves must become activated. Based on the “danger theory,” this occurs following the exposure of DCs to damage-associated molecular patterns, of which many have been proposed, including uric acid, heat-shock proteins (HSPs), and various cytokines [26].

It is important to note that antigen presentation may not occur after apoptotic damage because phagocytosis shields intracellular contents; moreover, if antigen presentation does occur, the lack of associated “danger” signals with apoptosis can lead to immune tolerance [27]. As such, the ratio of apoptosis to necrosis following thermal ablation is critical for subsequent acquired immune system activation.

## 4. Interactions between Thermal Ablation and the Immune System

A relationship between thermal therapies and the immune system has been recognized since the 1960s, when an antibody response was seen following cryotherapy in a rabbit model [28]. Of the existing thermal ablation techniques, the two modalities with the most well established immune interactions are RFA and cryoablation (Table 1). While HIFU [29, 30] and MWA have been shown to elicit an immune response, the magnitude of the response appears to be far greater with RFA and cryoablation [2].

### 4.1. Radiofrequency Ablation

**Figure 1:**
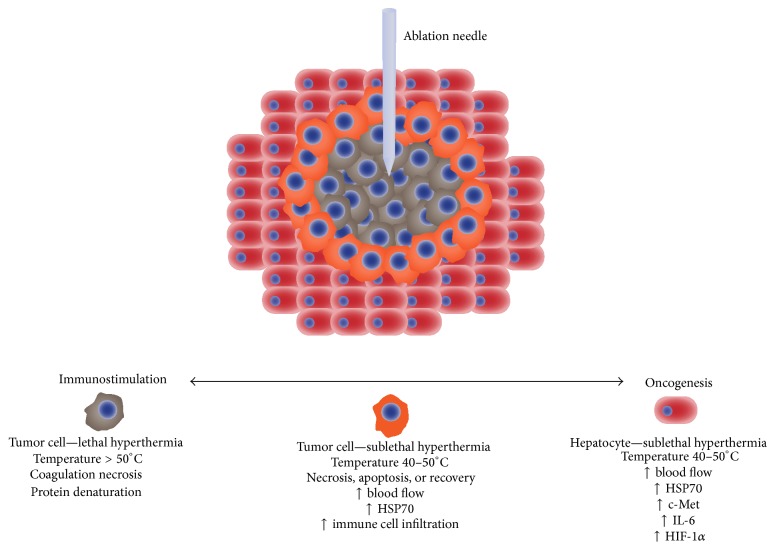
Thermal ablation and the proposed mechanisms for immunostimulation and oncogenesis in the liver. In the central heating zone, temperatures > 50°C cause coagulation necrosis. In the adjacent peripheral heating zone, lethal hyperthermia temperatures may not be achieved, leading to either necrosis, apoptosis, or recovery. In this zone, hyperemia results in increased oxygen delivery, and cell death results in the release of cytokines and other immune stimulatory factors such as heat-shock protein 70 (HSP70). As a result, either immunostimulation due to T-cell activation or immunosuppression due to T-cell anergy in the setting of apoptosis may occur. Sublethal thermal injury to the adjacent hepatocytes causes the release of additional growth factors such as c-Met that can cause systemic tumor growth stimulation.

Following heat-based ablation, numerous intracellular components that stimulate the innate immune system and can activate the acquired immune system are released (Figure 1). These include RNA, DNA, HSPs, and uric acid, as well as inflammatory cytokines such as interleukin-1*β* (IL-1*β*), IL-6, IL-8, and tumor necrosis factor-*α* (TNF-*α*) [31–34]. HSPs are common within tumor cells and are released during necrosis [35] as well as hyperthermia at 60°C [36]. Intracellularly, HSPs serve to prevent cell death by inhibiting apoptosis [36]. Once in the extracellular space, however, they drive the acquired immune response via several mechanisms, HSPs chaperone antigens to DCs for presentation, and they also function as danger signals to facilitate DC activation [35, 37–40]. HSP70 in particular has been implicated in the immune response to ablation therapy, and HSP70 levels are elevated in the serum of patients following RFA [41, 42].

RFA also reduces levels of regulatory T-cells (T_reg_) [32]. By suppressing these immunosuppressive cells, RFA may diminish immune tolerance to tumor cells, resulting in a more tumoricidal immune response. Indeed, levels of tumor-specific T-cells have been seen after RFA, and there is a survival benefit associated with higher levels of these cells [43, 44]. For example, intratumoral accumulation of CD8+ T-cells is associated with improved survival in patients with hepatocellular carcinoma who undergo resection surgery [45]. RFA has been shown to also result in an increase in tumor-specific antibodies, CD4+ cells, and CD8+ cells weeks to months after the ablation procedure [46].

On the other hand, since the early days of RFA, anecdotal reports of patients rapidly developing metastases following an ablation procedure have been described, and recently, an immunologic mechanism for these observations has begun to be elucidated. Indeed, RFA has been shown to cause distant tumor growth following hepatic ablation procedures in preclinical primary and metastatic liver cancer models [3, 47–50]. A key factor for these deleterious effects appears to be the response of the liver parenchyma that is included in the ablation zone. In those areas, elevated levels of HSPs, hypoxia induced factor-1*α* (HIF-1*α*), and other cytokines have been identified [34, 49, 51–53]. In an intriguing experiment, Ahmed et al. [47] performed RFA on a small portion of normal liver in a rat model and demonstrated accelerated growth in distant breast cancer xenografts compared to partial hepatectomy or sham surgery controls. This oncogenic response to RFA may be mediated by activation of hepatocyte regeneration signaling pathways by the heat-injured liver parenchyma, as inhibition of the hepatocyte growth factor/c-Met axis abrogates the accelerated tumor growth. Furthermore, IL-6 is an important driver of perilesional infiltration of immune cells: IL-6 knockout mice do not experience this infiltrative effect after ablation [3, 54]. Anti-IL-6 siRNA has been shown to suppress RFA-induced IL-6 production and its downstream oncogenic effects [54]. It is important to note, however, that while there is a growing body of preclinical data regarding the oncogenic impact of RFA, from a clinical standpoint, RFA has not been shown to worsen survival compared to untreated patients [48].

### 4.2. Cryoablation

The abscopal effect of cryotherapy has been reported as early as the 1970s [55, 56]. The concept of “cryoimmunology” originated in the 1960s when it was observed that serum anti-tumor antibodies develop after cryoablation [24, 57]. Anecdotal reports of the abscopal effect of cryotherapy in humans followed shortly thereafter in the 1970s [58]. Around the 1970s, it was also observed that cryotherapy can cause immunosuppression in rats. Leaving the bulk of the tumor in the animal was seen to result in slower tumor regression compared to regression after only a small amount of tissue remained [57]. Early studies also showed that cytotoxicity after cryoablation was tumor specific; that is, the lymphocytes harvested after cryoablation did not attack other tumor types. For example, cryoablation has been shown to confer resistance to rechallenge: in xenograft models, repeat delivery of tumor cells was less effective following cryoablation versus surgery performed on the initial tumor [57, 59–61]; this protection is tumor specific, as there was no prevention of tumor growth following challenge with another tumor cell line.

Cryoablation induces a much greater postablative immune response relative to RFA or MWA. This can be seen in greatly elevated levels of IL-1, IL-6, NF*κ*B, and TNF-*α* after cryoablation compared to the case after RF and MW [33, 34, 62, 63]. In comparative animal studies, the degree of DC antigen loading is greater with cryoablation versus RFA [9]. The proposed reason for this variation in immune activation is that hyperthermia based methods cause protein denaturation, reducing the number of intact antigens. Also, heat causes tissue coagulation and by doing so reduces the amount of intracellular contents that spill into circulation. Freezing, on the other hand, maintains cellular ultrastructure while increasing the permeability of plasma membranes. Also, it is for these reasons that we observe the phenomenon of cryoshock. Cryoablation causes the release of inflammatory intracellular debris, causing release of cytokines that can result in systemic inflammatory response syndrome (SIRS) [63]. A similar phenomenon is not observed in hyperthermia based modalities.

**Figure 2:**
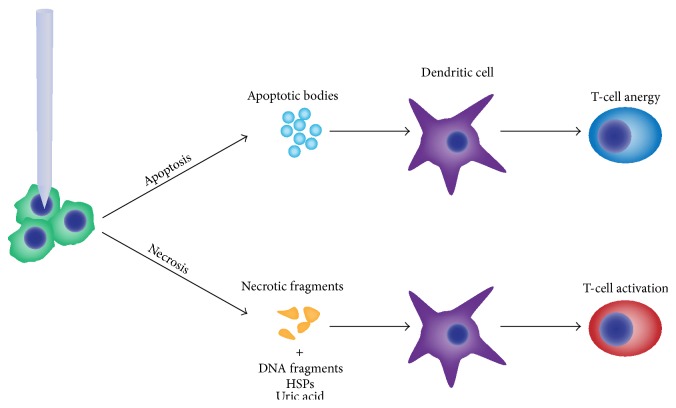
The method of tumor cell death following ablation plays a critical role in the downstream immunologic effects. Apoptosis results in the organized breakdown of dying cells into apoptotic bodies and does not release damage-associated molecular patterns such as DNA, HSPs, or uric acid as is seen with necrosis. Without the subsequent dendritic cell activation, T-cells do not receive costimulatory signals and therefore undergo anergy and clonal deletion.

However, the converse has also been seen, with no immune response or with immune suppression following cryoablation [64–66]. In rat fibrosarcoma models, increased susceptibility to rechallenge, increased primary tumor growth, and increased metastases have been seen following cryoablation [65, 67]. The balance between immunostimulation and immunosuppression may be related to technical factors during the cryoablation procedure. Across the multiple animal studies, there is substantial variation in the method of cryoablation performed, the minimum temperatures achieved, the duration of the therapy, and the number of freeze/thaw cycles performed. Indeed, the rate of freezing has been shown to impact immunostimulation [68]. These technical variations may translate into shifts in the relative balance of apoptosis and necrosis following ablation. It is difficult to predict whether apoptosis or necrosis is the dominant response after cryoablation [64, 68, 69], but this balance is of paramount importance, as dendritic cells that take up apoptotic cells do not mature, may have suppressed cytokine production, and may trigger clonal deletion and anergy (Figure 2). The target volume may play a key role in the subsequent immune response, potentially in a somewhat counterintuitive fashion. As shown by Blackwood and Cooper [57] and repeated by others [70], cryoablation zones that encompass the majority of a tumor may result in immunosuppression, while smaller volume ablations may result in immunostimulation and prolonged survival. Likewise, in an animal model of multiple hepatic metastases, cryoablation of multiple lesions was less effective than cryoablation of a single lesion at reducing the overall number of metastases [71].

## 5. Overview of Immune Checkpoint Therapy

The significant relationship between cancer and the immune system has been known for the last half century. The 1980s bore witness to an international effort to capitalize on this relationship through the development and application of tumor vaccines, an approach that, on the whole, categorically failed. In the past half decade, however, immunotherapy has been revived and is once again at the forefront of cancer therapies. The newest incarnation revolves around the manipulation of the regulatory systems that control T-cells. These immune checkpoint therapies are represented as major, disruptive breakthrough in cancer care [25]. Unlike other targeted therapies that focus on specific tumoral mutations, immune checkpoint drugs do not target tumor cells at all, but rather the immune cells that inhibit cytotoxic T-cell activity. While tumoral mutational status may vary not only over time but also within a lesion at single point in time, immune checkpoints are conserved and thus represent a stationary target.

Regulation of T-cell response is a highly complex system involving multiple cell types and multiple signaling pathways. At the time of T-cell activation, an inhibitory pathway is also turned on that will eventually suppress activation. This is done via the CTLA-4 surface marker on T-cells that, like CD28, binds B7 molecules on DCs, but at a higher affinity. Upon activation, T-cells begin to express CTLA-4, and as this molecule increasingly binds B7 complexes on the DC surface, costimulatory signals are lost, and the T-cell response is inactivated.

By inhibiting CTLA-4, in theory, one could persistently activate the cytotoxic T-cell response, in a manner that is independent of which tumor, or which antigen, has been targeted [25]. Ipilimumab is an anti-CTLA-4 antibody that has been shown to improve overall survival in melanoma patients [72, 73] in two Phase III trials. It has also shown clinical response in patients with renal, prostate, bladder, and ovarian cancers [74–78]. Ipilimumab is a groundbreaking approach to cancer therapy, and it opened the door for other immune “checkpoint” blockades, of which there are multiple. For example, another target is programmed cell death-1 (PD-1), a cell surface marker that is expressed by T-cells and interferes with signaling from the T-cell antigen receptor. PD-1 has two ligands, PD-L1 and PD-L2, both of which are expressed by many different cell types (unlike B7 which is expressed only by dendritic cells) including epithelial cells, endothelial cells, and tumor cells after exposure to interferon-*γ*. In this way, the PD-1/PD-L1 axis protects native host cells from T-cells. Anti-PD-L1 antibodies led to regression in melanoma, RCC, NSCLC, and bladder cancer [79, 80]. Anti-PD-1 drugs (nivolumab and pembrolizumab) are also effective across a range of malignancies [81].

## 6. Combining Thermal Ablation with Immune Therapy

The native immune response that follows an ablation procedure is unlikely to be of sufficient magnitude to cause system-wide, sustained regression of distant metastases [82]. However, a combinatorial approach that pairs ablation with immune stimulation may provide a synergistic effect. This approach has been tried in the preclinical setting, with early but promising results. Cryoablation has been combined with toll-like receptor activators [83–85] that stimulate DCs and thus promote cytotoxic T-cell activity. A greater antitumor response was seen following cryoablation when these drugs were administered compared to cryoablation alone. Additionally, the immune system can be “primed” following ablation by directly administering DCs into the ablation zone following cryotherapy. This has been associated with prolonged survival and reduced lung metastases in mice [82]. Dendritic cell based tumor vaccines have been combined with RFA [86], and dendritic cell loading after cryoablation may be more effective than after RFA [9]. Cryoablation used in conjunction with ipilimumab in a prostate cancer model slowed growth at secondary sites more effectively with cryoablation alone. Also, elevated levels of CD4+ and CD8+ T-cells were observed in distant sites [87].

Some clinical data combining ablation and immunotherapy are available as well. Cryotherapy in conjunction with GM-CSF (which promotes dendritic cell activation) has been seen to shrink metastases in lung cancer patients [88]. The same combination in pancreatic patients has been seen to increase survival [89].

## 7. Conclusions

The true extent of the systemic ramifications of thermal ablation is now beginning to be appreciated. What is clear is that thermal ablation cannot be considered solely as a locoregional therapy. The resultant inflammatory response, though at present limited and unpredictable, paves the way for an expanded role of thermal ablation as a stimulant to the immune system. Ablation, however, is a two-edged sword, and causes of and solutions to its oncogenic effects need to be investigated.

## Figures and Tables

**Table 1:** Selected publications surveying the clinical and preclinical evidence for immunomodulatory effects of RFA and cryoablation.

Modality	Cell type	Immune component	Species	Ref.
RFA				
*Potential tumor suppression*				
	VX2	↑ tumor-specific T-cells; ↑ T-cell infiltration	Rabbit	[44]
	HCC	DC activation; ↑ serum TNF-*α*, IL-1*β*	Human	[31]
	HCC	Tumor-specific CD4+ and CD8+ response	Human	[90]
	HCC	↑ HSP70 in tumor cell surface and cytoplasm	Human	[91]
	Primary and metastatic liver, kidney, and lung cancer	↑ HSP70	Human	[41]
	Primary and metastatic liver tumors	↑ memory T-cell trafficking, T-cell proliferation in metastatic cancer patients	Human	[92]
	HCC	↑ tumor-specific CD8+, correlating with progression-free survival after ablation	Human	[43]
	Primary and metastatic lung tumors	↓T_reg_; ↑ serum IL-8, IL-10, C3, C4, and CRP	Human	[32]
	Colon and kidney tumors and melanoma	↑ antigen-specific antibodies, CD4+ and CD8+ T-cells	Human	[46]
	HCC	↑ NK cell stimulation	Human	[93]
	Primary and metastatic colon, liver, kidney, and lung tumors, melanoma, and sarcoma	↑ serum IL-6, IL-10; ↔ serum TNF-*α*, IL-1*α*, and IL-2	Human	[33]
	Melanoma	Reduced tumor recurrence when combined with DC tumoral vaccine	Mouse	[86]
	Melanoma	DC activation; immunization against rechallenge with anti-CTLA-4 therapy	Mouse	[9, 94]
	Urothelial cancer	Tumor-specific T-cell activation, immunization against rechallenge	Mouse	[37]
	Hepatocytes	↑ IL-6	Rat	[34]
	Hepatocytes	↑ apoptosis, HSP70 in transition zone	Pig	[95]
*Potential tumor stimulation*				
	Hepatocytes	↑ intracellular HSP70 expression in tumor cells near blood vessels	Rat	[96]
	Hepatocytes (MDR2 knockout)	↑ tumor development, ↓ survival; effect diminished with c-Met inhibitor	Mouse	[3]
	Hepatocytes	↑ breast cancer xenograft growth; effect diminished with c-Met/VEGF inhibitors	Rat	[47]
	Colorectal	↑ hypoxia, HIF-1*α*, and HIF-2*α* in transition zone leading to tumor growth	Mouse/rat	[49]
	HCC	↑ HIF-1*α*, VEGF, and angiogenesis	Mouse	[51]
	Hepatocytes	↑ breast cancer xenograft growth; effect diminished with anti-IL-6 siRNA	Mouse/rat	[54]
Cryoablation				
*Potential tumor suppression*				
	Prostate	Remission of metastases following prostate cryoablation	Human	[55, 58]
	Sarcoma	Regression of remote tumor; immunization against rechallenge	Rat	[57]
	Breast	Tumor-specific T-cell response; immunization against rechallenge	Mouse	[61]
	Melanoma	Combination with TLR9 stimulation reduces local and remote tumors	Mouse	[83]
	Melanoma	DC activation; immunization against rechallenge with anti-CTLA-4 therapy	Mouse	[9]
	HCC	↑ IL-6, CRP, and IL-10; ↑ TNF-*α* and Th1/Th2 associated with remote tumor regression	Human	[97]
	Prostate	↑ TNF-*α*, IFN-*γ*, and Th1/Th2; ↑ tumor-specific T-cell response	Human	[98]
	Lung metastases	Combination with GM-CSF caused tumor-specific T-cell response and anti-tumor antibodies	Human	[88]
	Lung tumor, melanoma	Combination with DC therapy ↑ tumor-specific CD8+ T-cell response, ↑ survival	Mouse	[82]
	Colon	DC + Bacillus Calmette-Guérin cell wall skeleton caused tumor-specific CD8+ T-cell response and local and remote tumor regression	Mouse	[99]
*Potential tumor suppression*				
	Fibrosarcoma	↑ mortality from metastases compared to surgical excision; excision of cryoablated tumor reduced rate of metastasis	Rat	[64]
	Fibrosarcoma	↑ growth of pulmonary metastases after cryoablation of flank tumor	Rat	[65]
	Breast	Low freeze rate can ↑T_reg_,↑ remote metastases, and ↓ survival	Mouse	[68]

## References

[1] Wells S. A., Hinshaw J. L., Lubner M. G., Ziemlewicz T. J., Brace C. L., Lee F. T. (2015). Liver ablation: best practice. *Radiologic Clinics of North America*.

[2] Chu K. F., Dupuy D. E. (2014). Thermal ablation of tumours: biological mechanisms and advances in therapy. *Nature Reviews Cancer*.

[3] Rozenblum N., Zeira E., Scaiewicz V. (2015). Oncogenesis: an ‘off-target’ effect of radiofrequency ablation. *Radiology*.

[4] Nikfarjam M., Muralidharan V., Christophi C. (2005). Mechanisms of focal heat destruction of liver tumors. *Journal of Surgical Research*.

[5] Keisari Y. (2012). *Tumor Ablation*.

[6] Song C. W., Park H., Griffin R. J. (2001). Improvement of tumor oxygenation by mild hyperthermia. *Radiation Research*.

[7] Jones E. L., Zhao M.-J., Stevenson M. A., Calderwood S. K. (2004). The 70 kilodalton heat shock protein is an inhibitor of apoptosis in prostate cancer. *International Journal of Hyperthermia*.

[8] Tang D., Khaleque M. A., Jones E. L. (2005). Expression of heat shock proteins and heat shock protein messenger ribonucleic acid in human prostate carcinoma in vitro and in tumors in vivo. *Cell Stress and Chaperones*.

[9] den Brok M. H. M. G. M., Sutmuller R. P. M., Nierkens S. (2006). Efficient loading of dendritic cells following cryo and radiofrequency ablation in combination with immune modulation induces anti-tumour immunity. *British Journal of Cancer*.

[10] Ghanamah M., Berber E., Siperstein A. (2006). Pattern of carcinoembryonic antigen drop after laparoscopic radiofrequency ablation of liver metastasis from colorectal carcinoma. *Cancer*.

[11] Ohno T., Kawano K., Sasaki A., Aramaki M., Yoshida T., Kitano S. (2001). Expansion of an ablated site and induction of apoptosis after microwave coagulation therapy in rat liver. *Journal of Hepato-Biliary-Pancreatic Surgery*.

[12] Zitvogel L., Casares N., Péquignot M. O., Chaput N., Albert M. L., Kroemer G. (2004). Immune response against dying tumor cells. *Advances in Immunology*.

[13] Lu D. S. K., Raman S. S., Vodopich D. J., Wang M., Sayre J., Lassman C. (2002). Effect of vessel size on creation of hepatic radiofrequency lesions in pigs: assessment of the ‘heat sink’ effect. *American Journal of Roentgenology*.

[14] Lu D. S. K., Raman S. S., Limanond P. (2003). Influence of large peritumoral vessels on outcome of radiofrequency ablation of liver tumors. *Journal of Vascular and Interventional Radiology*.

[15] Thompson S. M., Callstrom M. R., Butters K. A. (2014). Heat stress induced cell death mechanisms in hepatocytes and hepatocellular carcinoma: in vitro and in vivo study. *Lasers in Surgery and Medicine*.

[16] Warters R. L., Roti Roti J. L. (1982). Hyperthermia and the cell nucleus. *Radiation Research*.

[17] Teng L.-S., Jin K.-T., Han N., Cao J. (2010). Radiofrequency ablation, heat shock protein 70 and potential anti-tumor immunity in hepatic and pancreatic cancers: a minireview. *Hepatobiliary and Pancreatic Diseases International*.

[18] Goldberg S. N., Ahmed M., Gazelle G. S. (2001). Radio-frequency thermal ablation with NaCl solution injection: effect of electrical conductivity on tissue heating and coagulation-phantom and porcine liver study. *Radiology*.

[19] Zhang L., Wang N., Shen Q., Cheng W., Qian G.-J. (2013). Therapeutic efficacy of percutaneous radiofrequency ablation versus microwave ablation for hepatocellular carcinoma. *PLoS ONE*.

[20] Lubner M. G., Brace C. L., Hinshaw J. L., Lee F. T. (2010). Microwave tumor ablation: mechanism of action, clinical results, and devices. *Journal of Vascular and Interventional Radiology*.

[21] Erinjeri J. P., Clark T. W. I. (2010). Cryoablation: mechanism of action and devices. *Journal of Vascular and Interventional Radiology*.

[22] Baust J. G., Gage A. A. (2005). The molecular basis of cryosurgery. *BJU International*.

[23] Gage A. A., Baust J. (1998). Mechanisms of tissue injury in cryosurgery. *Cryobiology*.

[24] Bastianpillai C., Petrides N., Shah T., Guillaumier S., Ahmed H. U., Arya M. (2015). Harnessing the immunomodulatory effect of thermal and non-thermal ablative therapies for cancer treatment. *Tumor Biology*.

[25] Sharma P., Allison J. P. (2015). The future of immune checkpoint therapy. *Science*.

[26] Pradeu T., Cooper E. L. (2012). The danger theory: 20 years later. *Frontiers in Immunology*.

[27] Ferguson T. A., Choi J., Green D. R. (2011). Armed response: how dying cells influence T-cell functions. *Immunological Reviews*.

[28] Yantorno C., Soanes W. A., Gonder M. J., Shulman S. (1967). Studies in cryo-immunology. I. The production of antibodies to urogenital tissue in consequence of freezing treatment. *Immunology*.

[29] Wu F., Zhou L., Chen W. R. (2007). Host antitumour immune responses to HIFU ablation. *International Journal of Hyperthermia*.

[30] Wu F., Wang Z.-B., Cao Y.-D. (2007). ‘Wide local ablation’ of localized breast cancer using high intensity focused ultrasound. *Journal of Surgical Oncology*.

[31] Ali M. Y., Grimm C. F., Ritter M. (2005). Activation of dendritic cells by local ablation of hepatocellular carcinoma. *Journal of Hepatology*.

[32] Fietta A. M., Morosini M., Passadore I. (2009). Systemic inflammatory response and downmodulation of peripheral CD25^+^Foxp3^+^ T-regulatory cells in patients undergoing radiofrequency thermal ablation for lung cancer. *Human Immunology*.

[33] Erinjeri J. P., Thomas C. T., Samoilia A. (2013). Image-guided thermal ablation of tumors increases the plasma level of interleukin-6 and interleukin-10. *Journal of Vascular and Interventional Radiology*.

[34] Ahmad F., Gravante G., Bhardwaj N. (2010). Changes in interleukin-1*β* and 6 after hepatic microwave tissue ablation compared with radiofrequency, cryotherapy and surgical resections. *American Journal of Surgery*.

[35] Chen T., Guo J., Han C., Yang M., Cao X. (2009). Heat shock protein 70, released from heat-stressed tumor cells, initiates antitumor immunity by inducing tumor cell chemokine production and activating dendritic cells via TLR4 pathway. *Journal of Immunology*.

[36] Schmitt E., Gehrmann M., Brunet M., Multhoff G., Garrido C. (2007). Intracellular and extracellular functions of heat shock proteins: repercussions in cancer therapy. *Journal of Leukocyte Biology*.

[37] Dromi S. A., Walsh M. P., Herby S. (2009). Radiofrequency ablation induces antigen-presenting cell infiltration and amplification of weak tumor-induced immunity. *Radiology*.

[38] Figueiredo C., Wittmann M., Wang D. (2009). Heat shock protein 70 (HSP70) induces cytotoxicity of T-helper cells. *Blood*.

[39] Tesniere A., Panaretakis T., Kepp O. (2008). Molecular characteristics of immunogenic cancer cell death. *Cell Death and Differentiation*.

[40] Multhoff G., Pockley A. G., Streffer C., Gaipl U. S. (2012). Dual role of heat shock proteins (HSPs) in anti-tumor immunity. *Current Molecular Medicine*.

[41] Haen S. P., Gouttefangeas C., Schmidt D. (2011). Elevated serum levels of heat shock protein 70 can be detected after radiofrequency ablation. *Cell Stress and Chaperones*.

[42] Kottke T., Sanchez-Perez L., Diaz R. M. (2007). Induction of hsp70-mediated Th17 autoimmunity can be exploited as immunotherapy for metastatic prostate cancer. *Cancer Research*.

[43] Hiroishi K., Eguchi J., Baba T. (2010). Strong CD8^+^ T-cell responses against tumor-associated antigens prolong the recurrence-free interval after tumor treatment in patients with hepatocellular carcinoma. *Journal of Gastroenterology*.

[44] Wissniowski T. T., Hünsler J., Neureiter D. (2003). Activation of tumor-specific T lymphocytes by radio-frequency ablation of the VX2 hepatoma in rabbits. *Cancer Research*.

[45] Unitt E., Marshall A., Gelson W. (2006). Tumour lymphocytic infiltrate and recurrence of hepatocellular carcinoma following liver transplantation. *Journal of Hepatology*.

[46] Widenmeyer M., Shebzukhov Y., Haen S. P. (2011). Analysis of tumor antigen-specific T cells and antibodies in cancer patients treated with radiofrequency ablation. *International Journal of Cancer*.

[47] Ahmed M., Kumar G., Moussa M. (2015). Hepatic radiofrequency ablation-induced stimulation of distant tumor growth is suppressed by c-Met inhibition. *Radiology*.

[48] Lencioni R., Cioni D., Crocetti L. (2005). Early-stage hepatocellular carcinoma in patients with cirrhosis: long-term results of percutaneous image-guided radiofrequency ablation. *Radiology*.

[49] Nijkamp M. W., van der Bilt J. D. W., de Bruijn M. T. (2009). Accelerated perinecrotic outgrowth of colorectal liver metastases following radiofrequency ablation is a hypoxia-driven phenomenon. *Annals of Surgery*.

[50] Nijkamp M. W., Borren A., Govaert K. M. (2010). Radiofrequency ablation of colorectal liver metastases induces an inflammatory response in distant hepatic metastases but not in local accelerated outgrowth. *Journal of Surgical Oncology*.

[51] Kong J., Kong J., Pan B. (2012). Insufficient radiofrequency ablation promotes angiogenesis of residual hepatocellular carcinoma via HIF-1*α*/VEGFA. *PloS ONE*.

[52] Solazzo S. A., Ahmed M., Schor-Bardach R. (2010). Liposomal doxorubicin increases radiofrequency ablation-induced tumor destruction by increasing cellular oxidative and nitrative stress and accelerating apoptotic pathways. *Radiology*.

[53] Yang W., Ahmed M., Tasawwar B. (2011). Radiofrequency ablation combined with liposomal quercetin to increase tumour destruction by modulation of heat shock protein production in a small animal model. *International Journal of Hyperthermia*.

[54] Ahmed M., Kumar G., Navarro G. (2015). Systemic siRNA nanoparticle-based drugs combined with radiofrequency ablation for cancer therapy. *PLoS ONE*.

[55] Ablin R. J., Soanes W. A., Gonder M. J. (1971). Prospects for cryo-immunotherapy in cases of metastasizing carcinoma of the prostate. *Cryobiology*.

[56] Gursel E., Roberts M., Veenema R. J. (1972). Regression of prostatic cancer following sequential cryotherapy to the prostate. *Journal of Urology*.

[57] Blackwood C. E., Cooper I. S. (1972). Response of experimental tumor systems to cryosurgery. *Cryobiology*.

[58] Soanes W. A., Ablin R. J., Gonder M. J. (1970). Remission of metastatic lesions following cryosurgery in prostatic cancer: immunologic considerations. *Journal of Urology*.

[59] Tanaka S. (1982). Immunological aspects of cryosurgery in general surgery. *Cryobiology*.

[60] Neel H. B., Ketcham A. S., Hammond W. G. (1973). Experimental evaluation of in situ oncocide for primary tumor therapy: comparison of tumor-specific immunity after complete excision, cryonecrosis and ligation. *Laryngoscope*.

[61] Sabel M. S., Nehs M. A., Su G., Lowler K. P., Ferrara J. L. M., Chang A. E. (2005). Immunologic response to cryoablation of breast cancer. *Breast Cancer Research and Treatment*.

[62] Jansen M. C., van Hillegersberg R., Schoots I. G. (2010). Cryoablation induces greater inflammatory and coagulative responses than radiofrequency ablation or laser induced thermotherapy in a rat liver model. *Surgery*.

[63] Chapman W. C., Debelak J. P., Pinson C. W. (2000). Hepatic cryoablation, but not radiofrequency ablation, results in lung inflammation. *Annals of Surgery*.

[64] Yamashita T., Hayakawa K., Hosokawa M. (1982). Enhanced tumor metastases in rats following cryosurgery of primary tumor. *Gan*.

[65] Shibata T., Yamashita T., Suzuki K. (1998). Enhancement of experimental pulmonary metastasis and inhibition of subcutaneously transplanted tumor growth following cryosurgery. *Anticancer Research*.

[66] Shibata T., Suzuki K., Yamashita T. (1998). Immunological analysis of enhanced spontaneous metastasis in WKA rats following cryosurgery. *Anticancer Research*.

[67] Hayakawa K., Yamashita T., Suzuki K. (1982). Comparative immunological studies in rats following cryosurgery and surgical excision of 3-methylcholanthrene-induced primary autochthonous tumors. *Gan*.

[68] Sabel M. S., Su G., Griffith K. A., Chang A. E. (2010). Rate of freeze alters the immunologic response after cryoablation of breast cancer. *Annals of Surgical Oncology*.

[69] Wing M. G., Rogers K., Jacob G., Rees R. C. (1988). Characterisation of suppressor cells generated following cryosurgery of an HSV-2-induced fibrosarcoma. *Cancer Immunology Immunotherapy*.

[70] Hanawa S. (1993). An experimental study on the induction of anti-tumor immunological activity after cryosurgery for liver carcinoma, and the effect of concomitant immunotherapy with OK432. *Nihon Geka Gakkai Zasshi*.

[71] Urano M., Tanaka C., Sugiyama Y., Miya K., Saji S. (2003). Antitumor effects of residual tumor after cryoablation: The combined effect of residual tumor and a protein-bound polysaccharide on multiple liver metastases in a murine model. *Cryobiology*.

[72] Robert C., Thomas L., Bondarenko I. (2011). Ipilimumab plus dacarbazine for previously untreated metastatic melanoma. *The New England Journal of Medicine*.

[73] Hodi F. S., O'Day S. J., McDermott D. F. (2010). Improved survival with ipilimumab in patients with metastatic melanoma. *The New England Journal of Medicine*.

[74] Weber J. S., O'Day S., Urba W. (2008). Phase I/II study of ipilimumab for patients with metastatic melanoma. *Journal of Clinical Oncology*.

[75] Yang J. C., Hughes M., Kammula U. (2007). Ipilimumab (anti-CTLA4 antibody) causes regression of metastatic renal cell cancer associated with enteritis and hypophysitis. *Journal of Immunotherapy*.

[76] van den Eertwegh A. J. M., Versluis J., van den Berg H. P. (2012). Combined immunotherapy with granulocyte-macrophage colony-stimulating factor-transduced allogeneic prostate cancer cells and ipilimumab in patients with metastatic castration-resistant prostate cancer: a phase 1 dose-escalation trial. *The Lancet Oncology*.

[77] Carthon B. C., Wolchok J. D., Yuan J. (2010). Preoperative CTLA-4 blockade: tolerability and immune monitoring in the setting of a presurgical clinical trial. *Clinical Cancer Research*.

[78] Hodi F. S., Butler M., Oble D. A. (2008). Immunologic and clinical effects of antibody blockade of cytotoxic T lymphocyte-associated antigen 4 in previously vaccinated cancer patients. *Proceedings of the National Academy of Sciences of the United States of America*.

[79] Brahmer J. R., Tykodi S. S., Chow L. Q. M. (2012). Safety and activity of anti-PD-L1 antibody in patients with advanced cancer. *The New England Journal of Medicine*.

[80] Powles T., Eder J. P., Fine G. D. (2014). MPDL3280A (anti-PD-L1) treatment leads to clinical activity in metastatic bladder cancer. *Nature*.

[81] Topalian S. L., Hodi F. S., Brahmer J. R. (2012). Safety, activity, and immune correlates of anti-PD-1 antibody in cancer. *The New England Journal of Medicine*.

[82] Machlenkin A., Goldberger O., Tirosh B. (2005). Combined dendritic cell cryotherapy of tumor induces systemic antimetastatic immunity. *Clinical Cancer Research*.

[83] den Brok M. H. M. G. M., Sutmuller R. P. M., Nierkens S. (2006). Synergy between in situ cryoablation and TLR9 stimulation results in a highly effective in vivo dendritic cell vaccine. *Cancer Research*.

[84] Redondo P., del Olmo J., López-Diaz de Cerio A. (2007). Imiquimod enhances the systemic immunity attained by local cryosurgery destruction of melanoma lesions. *Journal of Investigative Dermatology*.

[85] Nierkens S., den Brok M. H., Roelofsen T. (2009). Route of administration of the TLR9 agonist CpG critically determines the efficacy of cancer immunotherapy in mice. *PLoS ONE*.

[86] Liu Q., Zhai B., Yang W. (2009). Abrogation of local cancer recurrence after radiofrequency ablation by dendritic cell-based hyperthermic tumor vaccine. *Molecular Therapy*.

[87] Waitz R., Solomon S. B., Petre E. N. (2012). Potent induction of tumor immunity by combining tumor cryoablation with anti-CTLA-4 therapy. *Cancer Research*.

[88] Thakur A., Littrup P., Paul E. N., Adam B., Heilbrun L. K., Lum L. G. (2011). Induction of specific cellular and humoral responses against renal cell carcinoma after combination therapy with cryoablation and granulocyte-macrophage colony stimulating factor: a pilot study. *Journal of Immunotherapy*.

[89] Niu L., Chen J., He L. (2013). Combination treatment with comprehensive cryoablation and immunotherapy in metastatic pancreatic cancer. *Pancreas*.

[90] Zerbini A., Pilli M., Penna A. (2006). Radiofrequency thermal ablation of hepatocellular carcinoma liver nodules can activate and enhance tumor-specific T-cell responses. *Cancer Research*.

[91] Schueller G., Kettenbach J., Sedivy R. (2004). Heat shock protein expression induced by percutaneous radiofrequency ablation of hepatocellular carcinoma in vivo. *International Journal of Oncology*.

[92] Napoletano C., Taurino F., Biffoni M. (2008). RFA strongly modulates the immune system and anti-tumor immune responses in metastatic liver patients. *International Journal of Oncology*.

[93] Zerbini A., Pilli M., Laccabue D. (2010). Radiofrequency thermal ablation for hepatocellular carcinoma stimulates autologous NK-cell response. *Gastroenterology*.

[94] den Brok M. H. M. G. M., Sutmuller R. P. M., van der Voort R. (2004). In situ tumor ablation creates an antigen source for the generation of antitumor immunity. *Cancer Research*.

[95] Rai R., Richardson C., Flecknell P., Robertson H., Burt A., Manas D. M. (2005). Study of apoptosis and heat shock protein (HSP) expression in hepatocytes following radiofrequency ablation (RFA). *Journal of Surgical Research*.

[96] Bhardwaj N., Dormer J., Ahmad F. (2012). Heat shock protein 70 expression following hepatic radiofrequency ablation is affected by adjacent vasculature. *Journal of Surgical Research*.

[97] Osada S., Imai H., Tomita H. (2007). Serum cytokine levels in response to hepatic cryoablation. *Journal of Surgical Oncology*.

[98] Si T., Guo Z., Hao X. (2008). Immunologic response to primary cryoablation of high-risk prostate cancer. *Cryobiology*.

[99] Udagawa M., Kudo-Saito C., Hasegawa G. (2006). Enhancement of immunologic tumor regression by intratumoral administration of dendritic cells in combination with cryoablative tumor pretreatment and Bacillus Calmette-Guerin cell wall skeleton stimulation. *Clinical Cancer Research*.

